# Cathepsin Inhibition Modulates Metabolism and Polarization of Tumor-Associated Macrophages

**DOI:** 10.3390/cancers12092579

**Published:** 2020-09-10

**Authors:** Diana Oelschlaegel, Tommy Weiss Sadan, Seth Salpeter, Sebastian Krug, Galia Blum, Werner Schmitz, Almut Schulze, Patrick Michl

**Affiliations:** 1 Department of Internal Medicine I, Martin Luther University Halle-Wittenberg, 06120 Halle (Saale), Germany; diana.oelschlaegel@landw.uni-halle.de (D.O.); sebastian.krug@uk-halle.de (S.K.); 2 Institute for Drug Research, Hebrew University of Jerusalem, Jerusalem 9112102, Israel; tweisssadan@mgh.harvard.edu (T.W.S.); seth.salpeter@mail.huji.ac.il (S.S.); galiabl@ekmd.huji.ac.il (G.B.); 3 Department of Biochemistry and Molecular Biology, Theodor-Boveri-Institute, Biocenter, 97074 Würzburg, Germany; werner.schmitz@uni-wuerzburg.de (W.S.); almut.schulze@uni-wuerzburg.de (A.S.)

**Keywords:** cathepsin, activity-based probes, tumor-associated macrophage, autophagy, lysosome, lipid metabolism, inflammation

## Abstract

**Simple Summary:**

Stroma-infiltrating tumor-associated macrophages (TAM) play an important role in regulating tumor progression and chemoresistance. Many tumor-infiltrating macrophage populations can be identified by preferential expression of distinct marker genes associated with an M2 phenotype and may execute tumor-promoting functions by enhancing tissue remodeling, facilitating angiogenesis, and suppressing immune responses. In this study, we aimed to characterize the impact of cathepsins in maintaining the TAM phenotype. For this purpose, we investigated the molecular effects of cathepsin inhibition on the viability and polarization of human primary macrophages as well as its metabolic consequences. Pharmacological inhibition of cathepsins B, L, and S using a novel inhibitor, GB111-NH_2_, led to a polarization shift from M2- to M1 macrophages, associated with distinct alterations in lysosomal signaling and lipid metabolism. This could be therapeutically exploited in tumors with strong infiltration of M2-macrophages, thereby possibly reverting M2 polarization, overcoming drug resistance, and improving the prognosis of our patients.

**Abstract:**

Stroma-infiltrating immune cells, such as tumor-associated macrophages (TAM), play an important role in regulating tumor progression and chemoresistance. These effects are mostly conveyed by secreted mediators, among them several cathepsin proteases. In addition, increasing evidence suggests that stroma-infiltrating immune cells are able to induce profound metabolic changes within the tumor microenvironment. In this study, we aimed to characterize the impact of cathepsins in maintaining the TAM phenotype in more detail. For this purpose, we investigated the molecular effects of pharmacological cathepsin inhibition on the viability and polarization of human primary macrophages as well as its metabolic consequences. Pharmacological inhibition of cathepsins B, L, and S using a novel inhibitor, GB111-NH_2_, led to changes in cellular recycling processes characterized by an increased expression of autophagy- and lysosome-associated marker genes and reduced adenosine triphosphate (ATP) content. Decreased cathepsin activity in primary macrophages further led to distinct changes in fatty acid metabolites associated with increased expression of key modulators of fatty acid metabolism, such as fatty acid synthase (FASN) and acid ceramidase (ASAH1). The altered fatty acid profile was associated with an increased synthesis of the pro-inflammatory prostaglandin PGE_2_, which correlated with the upregulation of numerous NF_k_B-dependent pro-inflammatory mediators, including interleukin-1 (IL-1), interleukin-6 (IL-6), C-C motif chemokine ligand 2 (CCL2), and tumor necrosis factor-alpha (TNFα). Our data indicate a novel link between cathepsin activity and metabolic reprogramming in macrophages, demonstrated by a profound impact on autophagy and fatty acid metabolism, which facilitates a pro-inflammatory micromilieu generally associated with enhanced tumor elimination. These results provide a strong rationale for therapeutic cathepsin inhibition to overcome the tumor-promoting effects of the immune-evasive tumor micromilieu.

## 1. Introduction 

Infiltrating immune cells in the peritumoral stroma represent a characteristic feature of many solid tumors. Tumor-associated macrophages (TAM) are an essential constituent of the microenvironment in pancreatic cancer, both in the human disease and in genetic mouse models of pancreatic cancer [1,2]. TAM have been recognized as an important trigger of therapy resistance and are usually associated with poor prognosis [3,4]. Soluble factors secreted from infiltrating macrophages have been shown to facilitate cancer progression by mediating resistance to chemotherapeutic drugs, mediating tumor cell invasion, and fostering evasion of antitumor immune responses [5]. Cathepsins, which are expressed in TAM, have been shown to modulate the functional state of macrophages in various tumor models [6,7,8].

Macrophages are characterized by a high functional plasticity and acquire distinct, tissue-specific phenotypes in response to signals present within individual microenvironments [9,10]. In vitro, they can be polarized into opposing functional states, which have been named M1 and M2 polarizations in analogy to the T_H_1 and T_H_2 programming of adaptive immune cells. [11]. The M1 polarization can be induced by treatment with lipopolysaccharide (LPS) and interferon gamma (IFNγ) or tumor necrosis factor-alpha (TNF-α), leading to the expression of a pro-inflammatory cytokine pattern. In contrast, alternatively activated M2 macrophages can be polarized by stimulation with interleukin-4 (IL-4), IL-13, IL-10, or transforming growth factor-beta (TGF-β) [9,11]. M2 macrophages may execute tumor-promoting functions by enhancing tissue remodeling, facilitating angiogenesis, and suppressing immune responses [12,13]. The M1-M2 classification represents a highly simplified model for complex functional behavior and cellular plasticity. Still, many tumor-infiltrating macrophage populations can be identified by preferential expression of distinct marker genes associated with an M2 phenotype [14,15,16]. 

Various proteases, including cathepsins, are known as major determinants of the functional status of tumor-associated macrophages [8,17]. The cathepsin family of cysteine proteases contains 11 members which play an important role in protein breakdown in the lysosome, whose acidic environment provides the optimal conditions for their activity [18]. However, some cathepsins also show activity in the neutral pH range of the cytoplasm [18,19]. In addition, many cells, including macrophages, can also secrete cathepsins, which mediate effects in the microenvironment [20]. 

Lysosomes execute a variety of homeostatic functions, such as removal of damaged organelles and nutrient breakdown, thus providing substrates for resynthesizing of numerous compounds. [21]. This recycling process is known as autophagy [22]. During autophagy, cytoplasmic materials are separated into a double membrane vesicle (autophagosome) and delivered into the lysosomes, where a variety of hydrolases facilitate cargo degradation [21,23]. In addition to basal autophagy under normal physiological conditions, a large increase in autophagy rate can occur in response to stress signals, e.g., to prevent the accumulation of potentially cytotoxic proteins, to cover an increased energy demand, or to ensure a rapid synthesis of different effector molecules [24,25]. Thus, these reactions can contribute to the survival of the cell [26]. 

The process of autophagy, as well as the expression of key enzymes involved in it, are subject to a strictly regulated homeostasis. Dysregulation of the components can have far-reaching intra- and intercellular consequences [27]. TAM with distinct similarities to the anti-inflammatory, tumor-promoting M2 type typically secrete cathepsins, in particular cathepsin B, L, and S. These hydrolases are also expressed intracellularly at high levels [8,17,20]. However, the exact intracellular function of these cathepsins in TAM with respect to the formation or maintenance of the M2-like phenotype is largely unknown. 

In this study, we investigated the effect of targeted inhibition of cathepsin B, L, and S in human primary M2-polarized macrophages, representing an in vitro model for tumor-associated macrophages. Based on a strong expression of cathepsins B and L in distinct subsets of TAM, which we detected during carcinogenesis in both mouse models of pancreatic cancer and isolated pancreatic tissue of patients, we investigated whether pharmacological cathepsin inhibition leads to disruption or alteration in intracellular signaling pathways that are necessary for the preservation of the M2 phenotype. Our particular focus was to detect changes in lysosomal activity, autophagy, and energy metabolism, leading to functional alterations and changes in secreted effector cytokines. Our data demonstrate that cathepsin inhibition leads to profound alterations in autophagy and fatty acid metabolism and induces a shift towards a pro-inflammatory macrophage phenotype. 

## 2. Materials and Methods

### 2.1. Isolation of CD11b^+^ Macrophages from Murine Pancreas 

For isolation of pancreatic macrophages from wild-type mice, KC mice (LSL-Kras^G12/D+^; Pdx-1-Cre) and KPC mice (LSL-Kras^G12D/+^; LSL-Tpr53^R172H/+^; Pdx-1-Cre), animals were sacrificed at defined time points between 8–24 weeks of age. Tissues were digested with Collagenase A (Sigma-Aldrich, Darmstadt, Germany), followed by erythrocyte lysis and filtration through a 30 µm pore-size filter (Miltenyi Biotec, Bergisch Gladbach, Germany). CD11b^+^ macrophages were isolated by MACS MicroBeads (Miltenyi Biotec, Bergisch Gladbach, Germany) according to the manufacturer´s instructions. The purity of isolated cells was determined by flow cytometry. The sacrificing of the animals was approved within the central animal breeding authorization.

### 2.2. Isolation and Polarization of Human Monocytes

Human primary monocytes were obtained from blood samples from healthy donors. First, peripheral blood mononuclear cells (PBMCs) were isolated by separation over a Ficoll-Paque^TM^ gradient (GE Healthcare, Solingen, Germany). Subsequently, CD14 MicroBeads (Miltenyi Biotech, Bergisch Gladbach, Germany) were used for the magnetic separation of monocytes. The cells were cultured in a humidified atmosphere containing 5% CO_2_ at 37 °C. Monocytes were differentiated into macrophages over 7 days in RPMI media (Thermo Fisher Scientific, Dreieich, Germany) containing 5% fetal bovine serum (Capricorn Scientific, Ebsdorfergrund, Germany), 1 mM Non-Essential Amino Acids (Thermo Fisher Scientific, Dreieich, Germany), 2 mM L-Glutamine (Thermo Fisher Scientific, Dreieich, Germany), 1 mM Sodium Pyruvate (Sigma-Aldrich, Darmstadt, Germany), 1% Penicillin/Streptomycin (Thermo Fisher Scientific, Dreieich, Germany) and 100 ng/mL recombinant human macrophage colony-stimulating factor (M-CSF) (BioLegend, Koblenz, Germany) on Primaria^TM^ culture dishes (Thermo Fisher Scientific, Dreieich, Germany). Throughout the experiments, cells were maintained in the same media with 10 ng/mL of M-CSF. For polarization over 24 h into M1 or M2 type macrophages, the following cytokines were used: 20 ng/mL human Interferon γ (IFNγ), (Peprotech, Hamburg, Germany), 50 ng/mL Lipopolysaccharide, (LPS) from *Escherichia coli* (Sigma-Aldrich, Darmstadt, Germany), 20 ng/ml recombinant human IL-4 (Peprotech, Hamburg, Germany) and 20 ng/mL recombinant human IL-13 (Peprotech, Hamburg, Germany). This study was approved by Martin-Luther University Halle/Wittenberg (approval number 2015-106) on 22 January 2016.

### 2.3. Inhibition of Cathepsin Activity

GB111-NH_2_, a selective inhibitor for cathepsins B, L, and S activity, was used as described previously [28,29,30]. For experiments, cells were treated with GB111-NH_2_ (1 µm) for 24 h or Dimethyl sulfoxide (DMSO) (Sigma-Aldrich, Darmstadt, Germany) as a control before analysis.

### 2.4. Fluorescent Imaging and Immunoblotting

Cathepsin activity was determined by fluorescent imaging. Primary macrophages were cultured at a density of 4 × 10^6^ in 10 cm dishes. Following GB111-NH_2_ treatment, cells were treated with the fluorescent cathepsin activity-based probe GB123, as described previously [28,29,30], for 1 h followed by protein extraction in lysis buffer (50 mM TRIS-HCl pH 7.5, 150 mM NaCl, 0.1% SDS, 1% Sodium deoxycholate, and 1% Triton-X100). Ten micrograms of total proteins were suspended in Laemmli sample buffer and resolved on 12% SDS-PAGE. Gels were scanned by iNTAS advanced Imager with ChemoStar software (iNTAS Science Imaging, Göttingen, Germany) to detect the Cy5 fluorescent signal. For Western blots, protein extracts were prepared using lysis buffer as above, and 10 μg of total proteins were resolved on 10 or 12% SDS-PAGE. Proteins were then transferred onto a polyvinylidene difluoride (PVDF) membrane (GE Healthcare, Solingen, Germany) and incubated overnight at 4 °C with primary antibodies diluted in Tris-buffered saline with Tween20 (1× TBST) with 5% bovine serum albumin (BSA) (Sigma-Aldrich, Darmstadt, Germany) or 5% nonfat dry milk (Carl Roth, Karlsruhe, Germany) after blocking. For immunoblots the following antibodies were used: actin beta (Sigma-Aldrich, Darmstadt, Germany) 1:10,000; acid ceramidase (ASAH1), (Santa Cruz, Heidelberg, Germany) 1:500; autophagy related 16 like 1 (ATG16L1), (Cell Signaling, Frankfurt am Main, Germany) 1:1000; caspase-3 (incl. cleaved form) (Cell Signaling, Frankfurt am Main, Germany) 1:1000; cytosolic phospholipase A2 (cPLA2), (Santa Cruz, Heidelberg, Germany) 1:500; cathepsin B (CTSB), (Santa Cruz, Heidelberg, Germany) 1:1000; cathepsin D (CTSD) (Santa Cruz, Heidelberg, Germany) 1:1.000; cathepsin L (CTSL) (Abcam, Berlin, Germany) 1:500; cathepsin S (CTSS) (Bioss, Boston, MA, USA) 1:500 fatty acid synthase (FASN), (cell signaling, Frankfurt am Main, Germany ) 1:1000; lysosomal associated membrane protein 1 (LAMP-1), (cell signaling, Frankfurt am Main, Germany) 1:1000; microtubule associated protein 1 light chain 3 beta (LC3B), (cell signaling, Frankfurt am Main, Germany) 1:1000; lysosomal phospholipase A2 (lPLA_2_), (Santa Cruz, Heidelberg, Germany) 1:500; nuclear factor kappa B (NF_k_Bp65), (cell signaling, Frankfurt am Main, Germany) 1:1000; pNF_k_Bp65 (cell signaling, Frankfurt am Main, Germany) 1:1000; nitric oxide synthase 2 (NOS2), (Abcam, Berlin, Germany) 1:350; poly(ADP-ribose) polymerase 1 (PARP-1), (cell signaling, Frankfurt am Main, Germany) 1:1000; phosphoinositide kinase, FYVE-type zinc finger containing (PIKFYVE), (Santa Cruz, Heidelberg, Germany) 1:500; transcription factor EB (TFEB), (cell signaling, Frankfurt am Main, Germany) 1:1000; ATPase H+ transporting V1 subunit B1 (V-ATPase B1/2), (Santa Cruz, Heidelberg, Germany) 1:500. Membranes were then incubated with horseradish peroxidase (HRP)-conjugated secondary antibodies: rabbit anti-mouse (Sigma-Aldrich, Darmstadt, Germany) or donkey anti-rabbit (Amersham, Amersham, United Kingdom) at room temperature for 1 h. Chemiluminescent signal was generated using the SERVALight Helios or SERVALight Polaris WB Substrate Kit (Serva, Heidelberg, Germany). Images were taken by iNTAS advanced Imager as described above. In general, representative blot images from at least three independently repeated experiments are shown.

### 2.5. RNA Isolation, cDNA Synthesis, and Quantitative Real-Time PCR (qPCR) 

Human macrophages: Cells were lysed in Qiazol lysis buffer, and total RNA was isolated using the miRNeasy Mini Kit (Qiagen, Hilden, Germany) according to the manufacturer’s instructions. Extracted RNA was quantified by a spectrophotometric technique using a Nanodrop instrument (Thermo Fisher Scientific). cDNA synthesis was performed using an Omniscript RT Kit (Qiagen) according to the manufacturer’s instructions. Gene expression was analyzed using Luna Universal qPCR Master Mix (2×) (New England Biolabs, Frankfurt am Main, Germany) and the measurements were performed using the 7500 Real-Time PCR system (Applied Biosystems, Dreieich, Germany). Ribosomal protein RPLP0 was used as a housekeeping gene for data normalization. Murine macrophages: RNA isolation, cDNA synthesis, and pPCR was performed as described previously [31]. The sequences of all the primers used are listed in Supplementary Table S1.

### 2.6. Flow Cytometry Analysis of Murine CD11b^+^ Macrophages and Human CD14^+^ Monocytes

Isolated pancreatic macrophages (CD11b^+^ cells) from wild-type, KC, and KPC mice were stained for extracellular membrane proteins using anti-CD11b-APC (BioLegend, Koblenz, Germany), anti-CD204-RPE (Biozol, Eching, Germany), and anti-MHCII-PE/Cy7 (BioLegend, Koblenz, Germany) antibodies and analyzed with a BD LSR II flow cytometer and FlowJo software (Becton Dickinson, Heidelberg, Germany). For analysis of the purity of isolated human blood monocytes (CD14^+^ cells) an anti-CD14-FITC (Miltenyi Biotec) antibody was used. 

### 2.7. Immunofluorescence Microscopy 

For analysis of cathepsins B and L expression in combination with staining for the monocyte/macrophage marker CD68 in tissues, human pancreatic samples were provided by the Department of Pathology, Philipps-University Marburg, according to the guidelines of the local ethics committee. Tissue was sectioned using a microtome to 5 μM slices. The following primary antibodies were used: cathepsin B (Goat, R&D Systems, Wiesbaden, Germany, 1:100), cathepsin L (Goat, R and D Systems, Wiesbaden, Germany, 1:100), CD68 (Mouse, Cell Marque, Darmstadt, Germany, 1:100). Secondary antibodies were purchased from the Jackson Laboratory. Images were analyzed using a 20× objective on an Olympus FluoView FV10i (Miami, FL, USA) confocal laser scanning microscope. 

For immunofluorescence studies on cells, inhibitor-treated cells were fixed in 4% PFA on coverslips for 20 min. This was followed by a repeated wash step with 1× PBS. Cells were incubated for 30 min at RT with animal serum (Life Technologies, Dreieich, Germany) to block unspecific epitopes. After washing with PBS, cells were incubated with the primary antibodies for 1 h at 37 °C. Cells were washed 3 times with PBS and incubated in secondary antibodies for 1 h at 37 °C. Cells were washed again 3 times in PBS and incubated for 5 min with 4′,6-Diamidine-2′-phenylindole dihydrochloride (DAPI, Sigma-Aldrich, Darmstadt, Germany) for nucleic staining. Finally, cells were washed with water. The coverslips were fixed using Dako Mounting Medium (Dako, Waldbronn, Germany) as an embedding solution. The following primary and secondary antibodies were used: LC3B (Cell Signaling) 1:200, goat anti-rabbit IgG H&L Alexa Fluor^®^ 488 (Abcam, Berlin, Germany). 

An LYSO-ID^®^ Red Lysosomal Detection Kit (Enzo Life Sciences, Lörrach, Germany) was used as described in the manufacturer’s instructions. The slides were stored in the dark at RT until images were captured using a Biozero fluorescence microscope (Keyence, Neu-Isenburg, Germany) and analyzed using the equipped software BZ-Analyzer. All light microscopic images for evaluation of cell morphology after treatment with the cathepsin inhibitor GB111 were performed with the same microscope as described above.

### 2.8. Cell Viability Assay

As a method for adenosine triphosphate (ATP) quantification as an indicator of metabolically/active cells, the CellTiter-Glo^®^ Luminescent Cell Viability Assay (Promega, Walldorf, Germany) was used according to the manufacturer’s instructions. Primary macrophages (3 × 10^4^) were polarized in a 96-well opaque-walled plate in complete RPMI media and treated with GB111-NH_2_ as described above. After the addition of the CellTiter-Glo^®^ reagent followed by a 10-minute incubation step, the analysis was carried out using a LUMINOSCAN^TM^ ASCENT (Thermo Fisher Scientific, Dreieich, Germany).

### 2.9. Mass Spectrometry

For the mass spectrometric analyses, samples of 1 million cells each were used. The macrophages were polarized as described before and treated with the cathepsin inhibitor. DMSO-treated cells were used as a control.

Total Fatty Acid Determination Based on Bligh Dyer Extraction: Macrophage lipids were extracted according to the Bligh Dyer protocol [32]. Briefly, 200 µL of methanol (MeOH)/water (H_2_O) (4/1; *v*/*v*), 20 µL standard solution containing 5 mM each of tridecanoic acid, nonadecanoic acid, and Ibuprofen in MeOH/chloroform (CHCl_3_) (1/1; *v*/*v*) and 30 µL 0.2 M hydrochloric acid (HCl) was added to the macrophage cell pellets and mixed vigorously. Subsequently, 90 µL CHCl_3_, 100 µL CHCl_3_, and 100 µL H_2_O were added and mixed vigorously after each addition. The resulting suspension was centrifuged, and the lipid-containing lower phase was evaporated to dryness at 45 °C under a stream of nitrogen gas. The resulting lipid residue was hydrolyzed by heating for 1 h at 80 °C in 0.5 mL 0.3 M potassium hydroxide (KOH) in MeOH/H_2_O (9/1; *v*/*v*). Neutral lipids were extracted twice with 0.5 mL hexane each, and free fatty acids were extracted twice with 0.5 mL hexane each after acidification with 50 µL formic acid (HCOOH). The combined fatty acid extracts were evaporated at 45 °C under a stream of nitrogen gas. For mass spectrometric analysis, the fatty acids were dissolved in 1 mL acetonitrile (CH_3_CN)/H_2_O/HCOOH (90/9.9/0.1; *v*/*v*/*v*) and directly injected into the heated electrospray ionization (HESI) probe of the mass spectrometer at a flow rate of 10 µL/min. Each sample was measured three times for 0.5 min. using the following MS settings: Sheath gas, 6; spray voltage, 3.6 kV. The capillary temperature was set at 320 °C, and S-lens was 55. MS data were acquired at 70 k resolution in full MS negative mode with a scan range of 150–600 *m*/*z*, 1E6 AGC-target for a max. IT of 50 milliseconds. For data evaluation, intensities of signals corresponding to the calculated fatty acid masses (MIM-H^+^ ± 2 mMU) were accumulated for 0.5 min and analyzed using TraceFinder software (Thermo Fisher Scientific). Ultrapure water was obtained from a Millipore system (Milli-Q Merck Millipore, Darmstadt, Germany). Solvents, formic acid, and standard compounds were purchased from Merck. The equipment used for MS analysis was a Thermo Scientific Q exactive mass spectrometer (QE-MS) equipped with a HESI probe (Thermo Fisher Scientific, Dreieich, Germany).

Determination of Bis(monoacylglycero)phosphate (BMPs)/Phosphatidylglycerol (PGs) in Cell Extracts: Macrophage BMPs/PGs were extracted applying the BuMe method [33]. Briefly, the macrophage cell pellets were extracted with 400 µL n-butanol (BuOH)/MeOH (3/1, *v*/*v*) containing 0.1 µM 14:0/14:0-BMP, 300 µL heptane/ethylacetate (3/1; *v*/*v*) and 280 µL 1% formic acid (in water). After vigorous mixing, samples were centrifuged (2 min max. rpm). Liquid phases were separated, and the lower phase was re-extracted with 300 µL heptane/ethylacetate (3/1, *v*/*v*). The combined extracts were evaporated at 50 °C under a stream of nitrogen gas. For lipid class separation, the resulting lipid residue was resuspended in 150 µL hexane/acetic acid ethylester (4/1; *v*/*v*) and applied to a SiO_4_-column. Unwanted lipids were eluted by applying 750 µL hexane/acetic acid ethylester (9/4, *v*/*v*) and 100 µL MeOH. BMPs/PGs were eluted with 350 µL MeOH. The eluates were evaporated at 50 °C under a stream of nitrogen gas, and the resulting pellets were redissolved in 50 µL CH_3_CN/5 mM ammonium acetate (NH_4_OAc) (75/25, *v*/*v*). For LC/MS analysis, 5 µL of the resulting solution was applied to a hydrophilic interaction liquid chromatography (HILIC) column (at 30 °C) and eluted with an inverse CH_3_CN gradient: mobile phase A consisted of 5 mM NH_4_OAc in CH_3_CN/H_2_O (95/5, *v*/*v*), and mobile phase B consisted of 5 mM NH_4_OAc in CH_3_CN/H_2_O (5/95, *v*/*v*). After sample application, the LC gradient program was 0% solvent B for 1 min, followed by a linear increase to 60% solvent B within 5 min, then maintaining 60% B for 13 min, then returning to 0% B in 1 min and 5 min 0% solvent B for column equilibration before each injection. The flow rate was maintained at 0.35 mL/min. The eluent was directed to the HESI source of the QE-MS from 1.86 min to 7.0 min after sample injection. MS data were acquired at 70 k resolution in full MS negative mode with a scan range of 650–880 *m*/*z*, 1E6 AGC-target for a max. IT of 200 milliseconds. The fatty acid composition of isomeric BMPs/PGs was analyzed by fragmentation analysis in parallel reaction monitoring (PRM) mode (0.4 *m*/*z* isolation window, 2E4 AGC-target, NCE 30). HESI Source Parameters: sheath gas 30, aux gas 10, sweep gas 3, spray voltage 2.5 kV, capillary temp. 320 °C, S-lens RF level 55.0, and aux gas heater temp. 120 °C. For data evaluation, the area of peaks corresponding to the calculated BMP/PG masses (MIM-H^+^ ± 3 mMU) was integrated using TraceFinder software (Thermo Fisher Scientific).

HPLC–MS Solvents, LC–MS NH_4_OAc, and 14:0/14:0-BMP were purchased from Merck, SiO_4_-Matrix Columns: Strata SI-1 Silica (Phenomenex, Aschaffenburg, Germany). The equipment used for LC/MS analysis was a Thermo Scientific Dionex Ultimate 3000 UHPLC system hyphenated with a Q Exactive mass spectrometer (QE-MS) equipped with a HESI probe (Thermo Fisher Scientific, Dreieich, Germany). UPLC-precolumn: Acclaim Mixed-Mode HILIC column (5 μm particles, 10 × 2 mm) (Thermo Fisher Scientific, Dreieich, Germany). UPLC-column: Acclaim RSLC Mixed-Mode HILIC column (2.2 μm particles, 150 × 2.1 mm) (Thermo Fisher Scientific, Dreieich, Germany).

### 2.10. Enzyme-Linked Immunosorbent Assay (ELISA) of Prostaglandin E2 (PGE_2_)

The concentration of secreted PGE_2_ was analyzed in macrophage culture supernatant after GB111-NH_2_ treatment using a chemiluminescent based PGE_2_ Clia Kit (ENZO Life Sciences, Lörrach, Germany) according to the manufacturer’s instructions. The analysis was carried out on a microplate reader (TECAN, Crailsheim, Germany).

### 2.11. Statistical Analysis

Statistical analyses were performed in Graphpad Prism 5 (GraphPad Software, Inc., San Diego, CA, USA). Statistical significance was determined by a two-sided Student’s *t*-test. The statistical analyses were carried out in each case, with results from at least three independently repeated experiments. Data are presented as mean ± standard deviations (SD) and *p*-values below 0.05 were considered to be statistically significant (* *p* ≤ 0.05; ** *p* ≤ 0.01; *** *p* ≤ 0.001). 

## 3. Results

### 3.1. Cathepsins B and L Expression in Tumor-Infiltrating Macrophages Is Increased during Pancreatic Carcinogenesis

**Figure 1:**
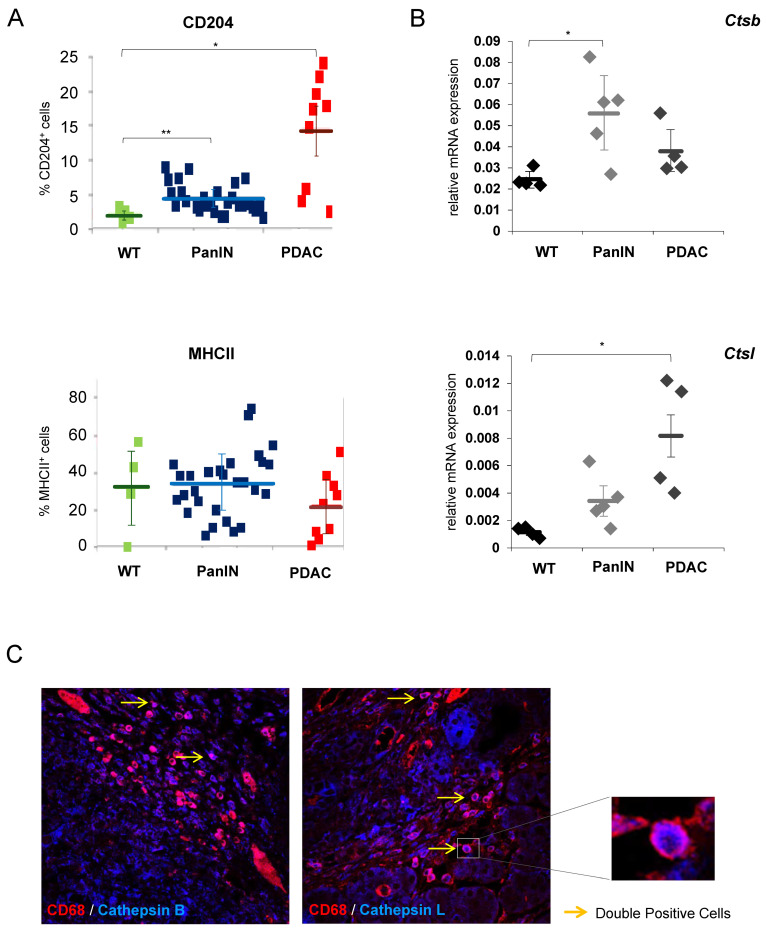
(**A**) During pancreatic carcinogenesis from pancreatic intraepithelial precursor (PanIN) lesions to invasive cancer (PDAC), isolated infiltrating murine CD11b^+^ cells showed an increase in CD204, whereas MHCII expression was not significantly affected. Cells from wild type mice were used as control. Data are presented as mean ± standard deviations (SD) and *p*-values below 0.05 were considered to be statistically significant (* *p* ≤ 0.05; ** *p* ≤ 0.01). (**B**) Changes in the expression profiles of cathepsin B and L were determined by real-time PCR in murine CD11b^+^ cells isolated from PanIN lesions and pancreatic ductal adenocarcinoma (PDAC) lesion from KC and KPC mice. Ribosomal protein RPLP0 was used as a housekeeping gene for data normalization. Cells from wild type mice were used as control. (**C**) Representative immunofluorescence pictures showing cathepsin B and L positivity of distinct subsets of tumor-associated macrophages in human pancreatic cancer tissues. A double-staining with CD68 and cathepsin B or cathepsin L antibodies was done, and representative double-positive cells are highlighted with yellow arrows.

To systematically analyze changes in expression patterns of cathepsin B and L in myeloid cell populations at different stages of carcinogenesis, we used both KC and KPC mice, with KC mice genetically and histologically recapitulating pre-invasive pancreatic intraepithelial precursor lesions (PanIN) and KPC mice genetically resembling invasive cancers that histologically evolve sequentially from pre-invasive PanIN lesions. Wild-type animals were used as controls. We isolated pancreatic CD11b+ myeloid cells from wild-type animals, KC mice with PanIN lesions, and KPC mice with invasive PDAC (pancreatic ductal adenocarcinoma). The purity of isolated myeloid cells was confirmed via FACS. We could confirm our previous results showing that during pancreatic carcinogenesis from PanIN lesions to invasive cancer, infiltrating CD11b^+^ cells exhibit an increase in the M2 markers CD204, whereas the M1 markers MHCII was not significantly affected (Figure 1A). [34]. In these isolated pancreatic CD11b^+^ cells, we could also detect an increase in cathepsin B (*Ctsb*) and L (*Ctsl*) mRNA during stepwise pancreatic carcinogenesis, reaching significance for cathepsin B in PanIN lesions and for cathepsin L in PDAC lesions, as compared to wild-type pancreata (Figure 1B). Cathepsin B and L positivity of distinct subsets of tumor-associated macrophages was also confirmed in human pancreatic cancer tissues using immunofluorescence double-staining with CD68 and cathepsin B or L antibodies, respectively (Figure 1C). 

These data indicate that both cathepsins B and L are upregulated in a distinct population of tumor-associated myeloid cells in murine and human tissues and that the expression of cathepsin L increases with disease progression. 

### 3.2. Effective Inhibition of Cathepsin B, L, and S Activity in Macrophages

**Figure 2:**
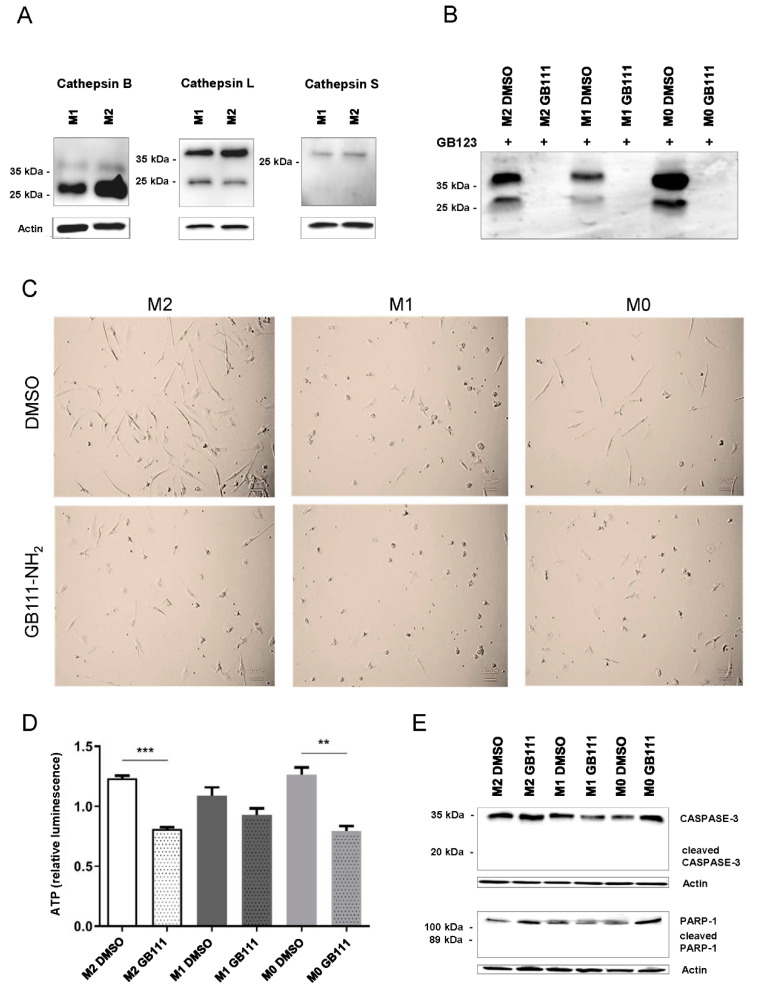
(**A**) Representative immunoblots of cathepsins B, L, and S, showing the general expression of these cathepsins both in polarized human pro-inflammatory M1 cells and in polarized anti-inflammatory M2 cells. Data are representative for at least three independent experiments, and ß-actin served as loading control. (**B**) Human primary macrophages were incubated with or without GB111-NH_2_ and labeled with GB123. The recovered lysates were separated in SDS PAGE, which was scanned by an iNTAS advanced Imager (Göttingen, Germany). The same amount of protein was loaded to the gel for all samples. The specific inhibitor GB111-NH_2_ efficiently blocked the cathepsin activity, indicated by the lack of a signal from the activity-based probe GB123. (**C**) The inhibitor GB111-NH_2_ causes morphological changes in human macrophages. While unpolarized M0 cells and polarized M2 cells were basically of a similar elongated shape, polarized M1 cells showed a round, spherical morphology. Incubation with GB111-NH_2_ leads in M0 and M2 macrophages to an approximation to the typical form of M1 cells. Representative images of cells derived from one donor are shown. (**D**) Cell viability assay for the quantification of adenosine triphosphate (ATP). The analysis of the ATP content allows conclusions to be drawn about metabolic and energy-consuming processes within the cells. M0, M1, and M2 macrophages were treated with DMSO or the inhibitor GB111-NH_2,_ as explained in the Methods section. Data are presented as mean ± standard deviations (SD) and *p*-values below 0.05 were considered to be statistically significant (** *p* ≤ 0.01; *** *p* ≤ 0.001). (**E**) Representative immunoblots of Caspase-3 and poly(ADP-ribose) polymerase 1 (PARP-1). The absence of the cleaved form of both proteins after inhibitor treatment illustrated that inhibition of the activity of cathepsins B, L, and S with GB111-NH_2_ did not initiate apoptosis in our study. Representative immunoblots which confirm the specificity of the used antibodies detecting Caspase-3 and PARP-1 including their respective cleaved forms, are shown in Supplementary Figure S1. Data are representative for at least three independent experiments, and ß-actin served as loading control.

To further study the functional impact of cathepsins, we next focused on human circulating monocytes obtained from healthy donors, which were differentiated into M1 and M2 macrophages in vitro. Expression of cathepsins B, L, and S protein could be detected both in the pro-inflammatory M1 population and the anti-inflammatory M2 cells (Figure 2A). Moreover, cathepsin activity could be clearly detected in all analyzed primary macrophage populations using an activity-based probe (GB123), which forms a covalent linkage to the target enzyme in an activity-dependent manner through a reactive moiety. Interestingly, cathepsin activity in M2 polarized cells and unpolarized cells (M0) was generally stronger than in M1 polarized cells (Figure 2B). Moreover, the specific cathepsin B, L, and S inhibitor GB111-NH_2_ [28,29] efficiently blocked cathepsin activity in all cell populations (Figure 2B). 

We next investigated the effect of cathepsin inhibition on the morphology of the different in vitro polarized macrophage populations. While M0 and M2-polarized primary macrophages exhibit morphological similarities, with cells appearing elongated representing a mesenchymal phenotype, M1-polarized macrophages generally show a round, spherical morphology. Incubation of M0 and M2 macrophages with the cathepsin inhibitor resulted in distinct morphological changes, resulting in a morphology that more resembled that of M1-polarized cells. In contrast, cathepsin inhibition did not alter the morphology of M1 polarized cells (Figure 2C).

To assess the impact of cathepsin inhibition on cell viability and metabolic activity, we measured cellular ATP content. In untreated cells of all macrophage populations, we generally observed slightly lower ATP levels in M1 macrophages compared to M0 and M2 macrophages (Figure 2D). After the treatment of the cells with the cathepsin inhibitor GB111-NH_2_, a decrease in the cellular ATP content was detected in all cell populations, reaching significance only in M0 and M2 macrophages (Figure 2D).

However, no signs of apoptosis were observed upon cathepsin inhibition, as shown by the absence of cleaved Caspase-3 and PARP-1 (Figure 2E).

### 3.3. Cathepsin Inhibition Leads to Disturbed Cellular Recycling Processes

**Figure 3:**
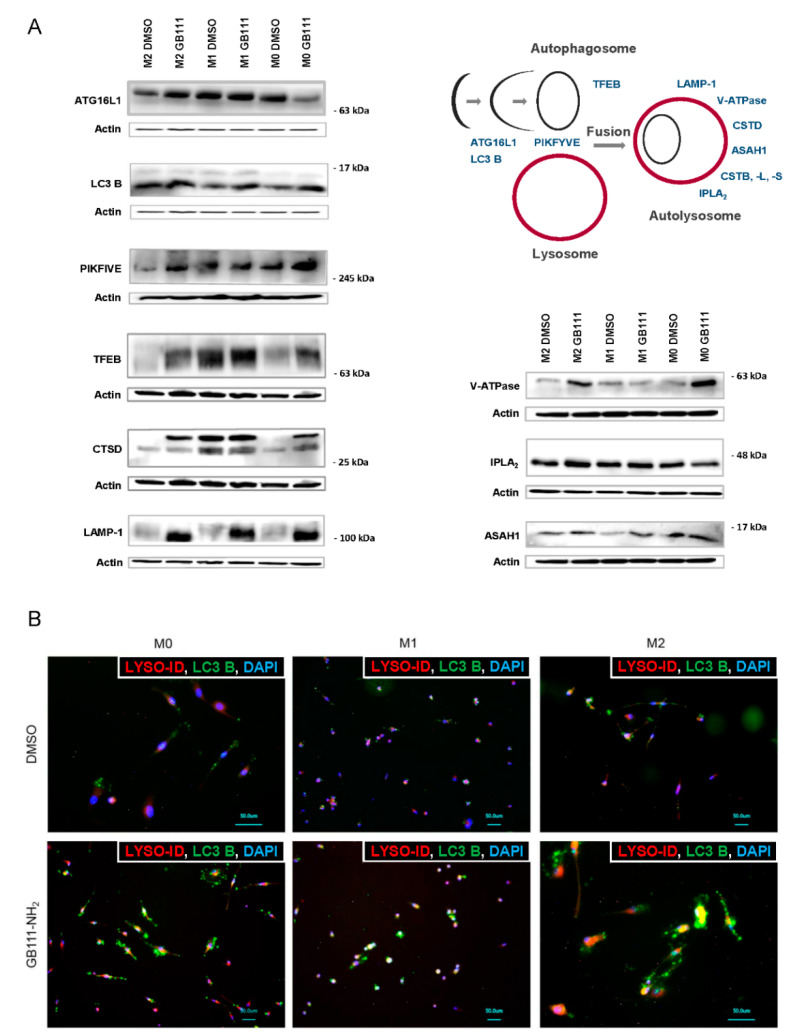
(**A**) Inhibition of cathepsin activity was accompanied by expression changes in typical autophagy- and lysosome-associated markers. The scheme illustrates the activity or localization of the analyzed markers. Human primary macrophages (M2, M1, and M0) were treated with GB111-NH_2_ or DMSO as described. Representative immunoblots detecting ATG16L1, LC3B, PIKFYVE, TFEB, CTSD, LAMP-1, V-ATPase, lPLA_2_, and ASAH1 are shown. Data are representative for at least three independent experiments, and ß-actin served as loading control. (**B**) Representative immunofluorescence images of LC3B (green), lyso-ID (red), and DAPI (blue) in M0, M1, and M2 macrophages treated with inhibitor GB111-NH_2_ or DMSO as described in the Methods section. The Lyso-ID^®^ Red Lysosomal Detection Kit (Enzo Life Sciences, Lörrach, Germany) was used for specific staining of lysosomes. The co-localization of autophagosome-associated LC3B and lyso-ID reflects the ability of the two organelles to fuse within the cells. DAPI (4′,6-Diamidin-2-phenylindol) was used for nuclear staining. Data are representative of at least three independent experiments. Scale bar is 50 μm.

We also observed that cathepsin B, L, and S inhibition was associated with an increase in several autophagy-related and lysosomal proteins, including ATG16L1, LC3B, PIKFYVE, TFEB, LAMP-1, V-ATPase, lPLA2, and ASAH1, suggesting changes in autophagy. This effect was particularly prominent in M2 polarized macrophages (Figure 3A). This accumulation of autophagy-related proteins could indicate a disturbed execution of the autophagy flux, as already demonstrated in other studies [35,36]. 

We also observed an increased co-localization of Lyso-ID, a lysosome specific dye, with LC3B. Beside the fusion of autophagosomes and lysosomes, this could indicate a possibly partial inhibition of the autophagy flux (Figure 3B). Interestingly, we saw that cathepsin B/L/S inhibition was also associated with an increased expression of cathepsin D, indicating a possible compensatory mechanism (Figure 3A).

### 3.4. Cathepsin Inhibition Correlates with Changes in the Cellular Fatty Acid and Phospholipid Profile

**Figure 4:**
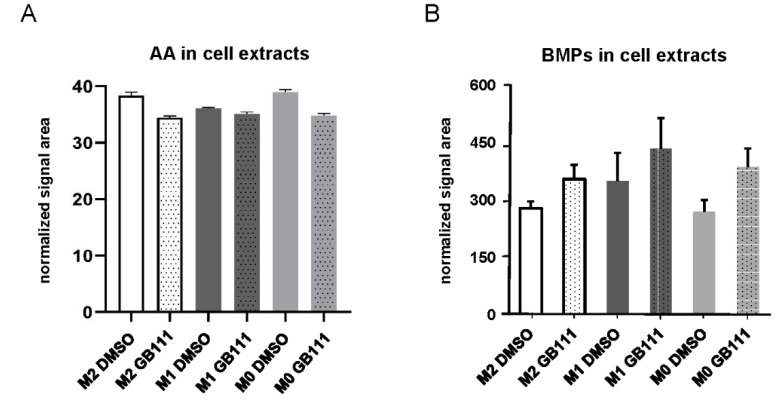
(**A**) Mass spectrometric analysis showing relative changes in arachidonic acid (AA) in cell lysates of human M0, M1, and M2 macrophages treated with GB111-NH_2_ or DMSO. Profiles of AA were normalized to the sum of all measured fatty acids signals (percent of total). Error bars represent SD of three technical replicates. (**B**) Mass spectrometric analysis showing changes in Bis(monoacylglycero)-phosphate (BMP) in the cell lysates of human M0, M1, and M2 macrophages treated with GB111-NH_2_ or DMSO. Data were normalized against the area of the signal of the externally added standard (BMP14:0/14:0). Error bars represent SD of three technical replicates.

Recent evidence has connected macrophage polarization with distinct metabolic alterations, including changes in lipid metabolism [37,38]. Therefore, we aimed to study the metabolic consequences of cathepsin B, L, and S inhibition in more detail, concentrating on potential changes in fatty acid metabolism. Mass spectrometry analyses revealed that treatment with GB111-NH_2_ leads to changes in the cellular fatty acid profile in all macrophage populations. These alterations were more pronounced in M0 and M2 polarized cells compared to the M1 population (Table S2). As the most prominent alteration induced by cathepsin inhibition, a reduction in the cellular content of arachidonic acid (AA) could be detected in the M2 and M0 populations (Figure 4A).

In addition, we use mass spectrometry to determine the level of Bis(monoacylglycero)-phosphate (BMP), a macrophage-relevant class of phospholipids specifically enriched in the membrane of intra-lysosomal vesicles, which have entered the lysosome via cellular recycling processes [39,40]. Changes in the cellular content of BMP can, thus, provide further evidence of disturbances in cellular autophagy processes as a result of cathepsin inhibition. We could detect an increase in BMPs, indicating an accumulation of lysosomes after inhibitor treatment (Figure 4B). 

### 3.5. Changes in the Fatty Acid Profile Are Associated with an Increased Synthesis of the Pro-Inflammatory Mediator Prostaglandin E2

Since AA serves as a substrate for the synthesis of the pro-inflammatory prostaglandin E_2_ (PGE_2_), we tested if changes in prostaglandin synthesis occurred as a result of the cathepsin inhibition. 

**Figure 5:**
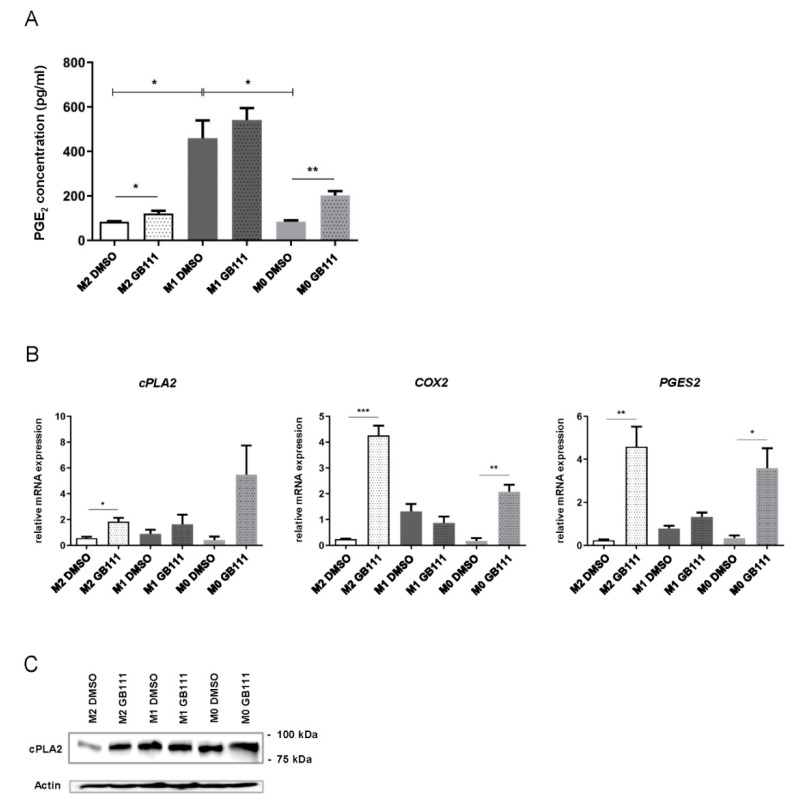
(**A**) Detection of prostaglandin E_2_ (PGE_2_) in the medium supernatant of human M0, M1, and M2 macrophages after inhibitor treatment using an enzyme-linked immunosorbent assay (ELISA). (**B**) Expression of mRNA levels of the PGE_2_ synthesis enzymes *cPLA2*, *COX-2,* and *PGES2* by real-time PCR in M0, M1, and M2 macrophages treated with GB111-NH_2_ or DMSO. Data are presented as mean ± standard deviations (SD) and *p*-values below 0.05 were considered to be statistically significant (* *p* ≤ 0.05; ** *p* ≤ 0.01; *** *p* ≤ 0.001). (**C**) Representative immunoblot showing cytosolic phospholipase A2 (cPLA2) protein in cells treated as in A. Data are representative for at least three independent experiments, and β-actin served as loading control.

As expected [41], the PGE_2_ concentration in the supernatant of pro-inflammatory M1 macrophages was significantly higher than in M0 and M2 macrophages. After cathepsin inhibition, however, a significant increase in the PGE_2_ concentration in the supernatant could be detected in both the M0 and M2 cells but not in the M1 population (Figure 5A). For PGE_2_ synthesis, membrane-bound AA is released and subsequently converted into PGE_2_ in a multi-step process involving various key enzymes [42]. These important enzymes include cPLA2, COX-2, and Prostaglandin E Synthase 2 (PGES2). We, therefore, measured the mRNA expression levels of these three enzymes. Inhibition of cathepsin activity in M2 macrophages caused a significant increase in the mRNA expression levels of *cPLA2*, *COX-2,* and *PGES2* (Figure 5B). In M0 macrophages, a similar trend was seen, reaching significance only for *COX-2* and *PGES2*. In contrast, the incubation of M1 cells with GB111-NH_2_ did not lead to a marked change in the expression of the enzymes (Figure 5B). In addition, cathepsin inhibition was associated with an increased expression of cPLA2 protein in M2 polarized macrophages (Figure 5C).

### 3.6. Cathepsin Inhibition Is Associated with an Enhanced Expression of Pro-Inflammatory and Fatty Acid Metabolism-Associated Genes

Since pharmacological cathepsin B, L, and S inhibition in M2 polarized macrophages revealed distinct morphological changes, increased lysosomal activity, and PGE_2_ synthesis, which are generally associated with the M1 phenotype, we hypothesized that cathepsin inhibition shifts macrophage polarization towards a more M1-like phenotype. To verify this hypothesis, we examined changes in the expression of a panel of genes associated with either the M1 or the M2 polarized phenotypes. This panel included several cytokines and effector proteins, such as *TNFα*, *IL-1a*, *IL-1b*, *IL-6*, *CCL2* (C-C motif chemokine ligand 2), and *NOS2* (nitric oxide synthase 2), as well as surface markers, like *CCR7* (C-C motif chemokine receptor 7) and *CD206* (mannose receptor C-type 1). Since fatty acid synthesis is known to play an important role in energy metabolism in pro-inflammatory M1 macrophages [43,44], we also analyzed the expression of *FASN* (fatty acid synthase), the enzyme catalyzing the synthesis of palmitate from acetyl-CoA and malonyl-CoA. In addition, we determined the expression of polarization-associated proteins by Western blot, including FASN, NOS2, NF_k_B (nuclear factor kappa B p65), and its phosphorylated form (pNFκBp65). 

**Figure 6:**
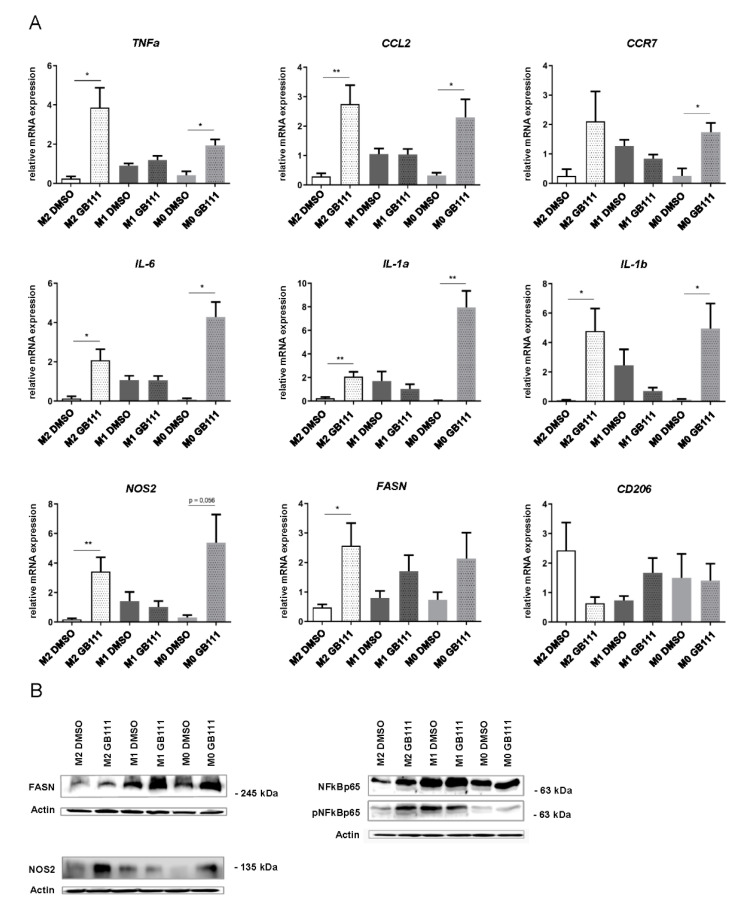
(**A**) Inhibition of cathepsin B, L, and S activity correlates with changes in the expression profiles of the M1 associated marker genes *TNFα*, *CCL2*, *CCR7*, *IL-6*, *IL-1a*, *IL-1b*, *NOS2,* and *FASN*, as well as the M2 marker *CD206*, as determined by real-time PCR. Data are presented as mean ± standard deviations (SD) and *p*-values below 0.05 were considered to be statistically significant (* *p* ≤ 0.05; ** *p* ≤ 0.01. (**B**) Representative immunoblots showing the expression of the M1 associated markers fatty acid synthase (FASN), nitric oxide synthase 2 (NOS2), and the detection of phosphorylated and total NFkBp65. Data are representative for at least three independent experiments, and β-actin served as loading control.

Overall, pharmacological inhibition of cathepsin B, L, and S led to a marked increase in pro-inflammatory mediators, especially in the M2 macrophages, and, to a lesser extent, in unpolarized M0 cells (Figure 6A). M1 macrophages generally showed a stronger baseline expression of pro-inflammatory markers without notable changes upon cathepsin inhibition. In line with these findings, there was a trend towards reduction in the typical M2 marker CD206 upon cathepsin inhibition; however, without reaching significance (Figure 6A). Similarly, cathepsin inhibition was associated with increased protein expression of FASN, NOS2, and pNFkBp65, especially in the M2 macrophages, also indicating a shift to M1 polarization (Figure 6B). 

## 4. Discussion

Infiltration of immune cells into the tumor-surrounding stroma can be observed in many solid tumors. Myeloid cells differentiating into tumor-associated macrophages (TAM) represent a crucial immune cell population within the tumor stroma. Dense TAM infiltration of the tumor microenvironment is usually associated with poor prognosis and increased resistance to chemotherapy, primarily due to a multitude of TAM-derived secreted mediators known to facilitate invasiveness, tumor cell survival, and immune evasion [45,46,47]. These macrophage-derived effector molecules include secreted cathepsins, such as cathepsin B, L, and S, which are known to contribute to the degradation of extracellular matrix compounds. Secreted cathepsins may also alter cell adhesive and migratory properties by modulating integrin receptor interactions with the extracellular matrix and also modulate cell trafficking and recruitment through cleavage of chemokines [47].

In addition to their extracellular function, cathepsins may also exert distinct intracellular effects. To date, however, the role of cathepsins in the formation and maintenance of the TAM phenotype remains to be fully elucidated. Many members of the cathepsin family are ubiquitously expressed in human tissues, with considerable overlap in the substrate specificity [18,48]. Cathepsins generally play an important role in numerous cellular processes, such as activation of protein precursors, regulation of differentiation pathways, cell cycle control, inflammatory signaling, and apoptosis [20,49]. In addition to their preferential localization in the acidic milieu of the lysosomes, cathepsin L and B can also be detected in the nucleus where they are involved in the regulation of cell cycle progression and histone processing [50,51,52,53]. Cathepsins are also involved in intracellular signaling pathways. It could be shown that cathepsin X disconnects mitogen-activated protein kinase (MAPK)/extracellular-signal-regulated kinase (ERK) and phosphoinositide 3-kinase (PI3K)/serine/threonine kinase (AKT) survival signaling in neuronal-like cells [47]. For various intracellular cathepsins, partially overlapping functions in the area of inflammasome activation have also been demonstrated [54]. The expression of distinct members of the cathepsin family is detectable in both M1 and M2 macrophages, playing an important role, particularly in the context of lysosomal recycling [35]. 

Many reports investigating the role of tumor-associated macrophages have utilized murine bone marrow-derived myeloid cells. Since considerable functional and phenotypic differences between murine and human innate immune cells have been reported [55], we focused on human primary myeloid cells. After in vitro polarization into the M2 macrophages, these cells represent a suitable human in vitro model for tumor-associated macrophages. We used the selective inhibitor GB111-NH_2_, which we have characterized previously [28], to inhibit the activity of cathepsin B, L, and S and investigated the consequences on the viability, metabolism, and polarization of the macrophages.

We were able to show that cathepsin inhibition in M2 macrophages leads to marked morphological changes from an elongated to a more rounded cell shape, typical of M1 macrophages. Various studies have already shown that macrophages undergo morphological changes in response to various soluble factors, demonstrating their overall high plasticity. Under the influence of pro-inflammatory M1-associated effectors, such as LPS and IFNγ, a compact, round cell shape is formed, while stimulation with M2-like cytokines, such as IL-4 or IL-13, results in an elongated morphology showing podosome-like protrusions [56,57,58]. This correlates with differences in actin organization between M1 and M2 macrophages [56,59]. While M1 cells have more clustered F-actin structures around the nuclei, M2 cells show more diffuse actin organization, holding a better potential for lamellipodia formation [57]. This M2-like phenotype was reverted upon cathepsin inhibition into an M1-like cell shape. 

In contrast to studies on murine primary macrophages that observed a marked induction of apoptosis following treatment with GB111-NH_2_, we found no evidence for apoptotic cell death or toxicity in human primary macrophages [28,60]. It cannot be ruled out that there are differences in the inhibitor uptake capacity and also the apoptosis machinery or other compensatory mechanisms between species.

A total of 11 different human cathepsins have been described. Among them, cathepsin B, L, and S have been predominantly associated with tumor progression in various cancer entities, including pancreatic ductal adenocarcinoma [49,61,62]. The inhibitor used in our study selectively inhibits the activity of cathepsin B, L, and S, leaving the activity of the remaining cathepsins unaffected [28,28,29]. In this context, we observed an increase in cathepsin D levels upon inhibitor treatment. It can be speculated that this most likely represents a compensatory upregulation. In line with this hypothesis, cleavage-site prediction models indicate overlapping substrate spectra among individual cathepsins including cathepsin B, D, S, and L [48]. It remains to be elucidated if, in addition to cathepsin D, other cathepsin family members are also upregulated as compensatory mechanism upon inhibition of cathepsin B, S, and L. 

Various studies observed changes in autophagy-related signaling cascades and lysosomal function following the inhibition of cathepsins. However, the functional consequences vary depending on the cellular context and range from increased autophagy, changes in the spectrum of recycled substrates to disruption of the entire process [28,63,64,65,66]. Our data detecting complex changes in the cellular recycling machinery are in line with several reports in the literature: Increased expression of the autophagy-associated transcription factor TFEB could also be detected in CTSB-deficient murine bone marrow-derived macrophages [67]. Furthermore, targeted inhibition of CTSL in a human neuroblastoma cell line blocked activation of caspase-3 and PARP-1 [68], similarly to our observations in human primary macrophages, in which inhibition of CTSL influenced autophagy but was unable to induce apoptosis [66]. Our results indicate that inactivation of cathepsin B, L, and S activity leads to increased expression of autophagy-associated markers, such as LC3B, ATG16L, TFEB, and PIKFYVE. These proteins exert key roles in the formation of autophagosomes and the fusion with lysosomes [69,70]. The activation of the transcription factor TFEB facilitates lysosome formation and has been associated with the regulation of the innate immune response in activated macrophages [71]. In addition, several proteins responsible for maintaining lysosomal integrity and pH homeostasis, such as LAMP-1 and V-ATPase [72,73], as well as lysosomal enzymes involved in the recycling of complex lipid structures and provision of fatty acids, such as lysosomal phospholipase A2 (lPLA_2_) and acid ceramidase (ASAH1) were upregulated upon cathepsin inhibition [74,75,76]. Furthermore, we were able to detect an increased content of cellular BMP upon cathepsin inhibition, which is known to play an important role in macrophages, especially with regard to the lysosomal recycling processes [39,40]. It is conceivable that the detected increased expression of some autophagy- and lysosome-associated proteins, as well as the phospholipid BMP, represents an accumulation due to a disturbed autophagy flux by inhibition of the cathepsins B, L, and S. It is possible that cells activate additional compensatory mechanisms including upregulation of other proteases, such as cathepsin D, thereby preventing a complete lysosomal recycling blockade. This could be a reason for the lack of apoptosis after inhibitor treatment. 

In order to investigate the effects of cathepsins B, L, and S inhibition on metabolic processes in macrophages, we first measured the cellular ATP content. Polarized M2 macrophages predominantly use oxidative phosphorylation for energy production, whereas glycolysis is more prominent in M1 macrophages [77]. Glycolysis generates a markedly lower ATP yield but permits a very rapid supply of energy required for important processes, such as phagocytosis [78]. Our results showed a reduction in the cellular ATP content upon cathepsin inhibition in M2 and M0 macrophages to levels comparable to M1 cells. It may be speculated that this is at least partly due to enhanced glycolytic activity. In line with these data, a recently published study in murine embryonic fibroblasts also revealed that cathepsin L inhibition led to a shift in cellular energy production towards glycolysis [79]. 

Recent data suggest that lipid metabolism may play an important role in polarization and phenotypic alterations of macrophages. In this context, the lipid composition in membranes affects the ability of phagocytosis, a key feature of M1 macrophages. Additionally, a high activity of enzymes, such as COX-2, cPLA2, and PGES2, has been reported in M1 cells, which leads to the release of arachidonic acid (AA) from phospholipids and ultimately promotes the stepwise synthesis of pro-inflammatory effectors, such as prostaglandins [80,81]. We performed mass spectrometric analyses to explore the potential impact of cathepsin inhibition on lipid metabolism in human primary macrophages. These experiments revealed distinct changes in the cellular fatty acid profile, being more pronounced in M0 and M2 polarized cells than in M1 macrophages. The most prominent changes caused by cathepsin inhibition were reductions in arachidonic acid levels. The vast majority of cellular AA is normally membrane-bound [82], which can be subsequently converted into the pro-inflammatory prostaglandin E2 (PGE_2_). In line with this, we detected increased expression levels of key enzymes for PGE_2_ synthesis, such as cPLA2, COX-2, and PGES2. The decreased overall content of AA in the cell, thus, most likely reflects an increased turnover and conversion of AA to PGE_2_. 

Enhanced glycolytic metabolism and synthesis of pro-inflammatory PGE_2_ represent typical M1-associated features, suggesting a shift from M2 to M1-like features upon cathepsin inhibition. We could corroborate this hypothesis by detecting an increased expression of several typical M1 mediators, including IL-1, IL-6, CCL2, TNFA, NOS2, NFkBp65, CCR7, and FASN, upon cathepsin inhibition in M2 and also—to a lesser extent—in M0 macrophages. 

Several studies have linked different cathepsins, including B, C, L, S, X, and Z, with pro-inflammatory processes in immune cells, including activation of the NLRP3-inflammasome [54,83]. Therefore, it appears likely that the upregulation of pro-inflammatory cytokines seen in our study after pharmacological inhibition of cathepsin B, S, and L is also at least in part due to compensatory upregulation of various other cathepsins, such as cathepsin D, known to contribute to the development and maintenance of a pro-inflammatory M1-like phenotype [84,85]. In this context, knockout experiments in murine primary microglia cells have demonstrated that cathepsin D plays an important role in the activation of NF_k_B-mediated signaling pathways [86]. Another cathepsin family member warranting investigation in this context is cathepsin C. Strong expression of cathepsin C was detected in murine M1 macrophages and seems to be important for M1 polarization status. Cathepsin C activity was reported to be associated with pro-inflammatory activation of the focal adhesion kinase (FAK) and p38MAPK/NFκB signaling [87]. 

In conclusion, targeted inhibition of cathepsins B, L, and S in human M2 macrophages is associated with far-reaching alterations in cellular signaling and metabolism, which leads to the acquisition of an M1-like pro-inflammatory phenotype. From a translational point of view, re-polarization from M2 towards an M1-like phenotype offers great potential for cancer therapy. Further studies are warranted to delineate the functional impact of cathepsin inhibition of phagocytosis, antigen presentation, and tumor cell cytotoxicity of tumor-associated macrophages in vitro and in vivo. Using a novel pharmacological approach for cathepsin inhibition, we cannot fully rule out that potential off-target pro-inflammatory effects are partly responsible for the observed effects. However, our results demonstrating changes in lysosomal activity, fatty acid metabolism, and synthesis of pro-inflammatory mediators, point consistently towards a shift to an M1-like phenotype. Simultaneous genetic knockdown or knockout strategies of all three cathepsins would be necessary to unequivocally rule-out potential off-target effects. 

## 5. Conclusions

In summary, our study provides preclinical evidence that pharmacological inhibition of cathepsin B, S, and L leads to a polarization shift from M2- to M1 macrophages, associated with distinct alterations in lysosomal signaling and lipid metabolism. This could be therapeutically exploited in tumors with strong infiltration of M2-macrophages, thereby possibly reverting M2 polarization, overcoming drug resistance, and improving the prognosis of our patients. 

## Supplementary Materials

The following are available online at https://www.mdpi.com/2072-6694/12/9/2579/s1, Figure S1. These immunoblots confirm the specificity of the used antibodies detecting Caspase-3 and PARP-1, including their respective cleaved forms, Table S1. List of all primer pairs used for real-time PCR analysis with human and murine cells, Table S2: Raw data of mass spectrometry analysis to determine the content of arachidonic acid and BMP in human macrophages.
