# Supplementary material for: Cathepsin Inhibition Modulates Metabolism and Polarization of Tumor-Associated Macrophages
Source: Cancers (Basel). 2020 Sep 10;12(9):2579. doi: 10.3390/cancers12092579 (PMC7563557; doi:10.3390/cancers12092579)
Supplement: Supplementary file 1 [file cancers-12-02579-s001.zip › cancers-874090-supplementary/cancers-874090-suppl_final.docx]

**Supplementary Materials**


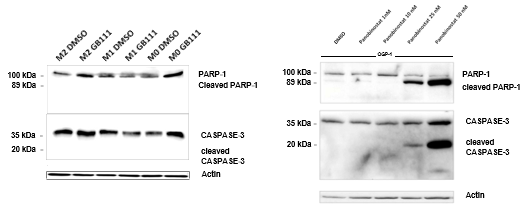


**Figure S1.** These immunoblots confirm the specificity of the used antibodies detecting Caspase-3 and PARP-1, including their respective cleaved forms*.* Shown are representative blots from a human pancreatic carcinoma cell line of islet cell origin (QGP*-*1)*.* Cells treated with different concentrations of Panobinostat (Histone deacetylase inhibitor, diluted in DMSO) are displayed in comparison to a control group (DMSO). Immunoblotting procedure was done as described and ß-actin served as loading control.


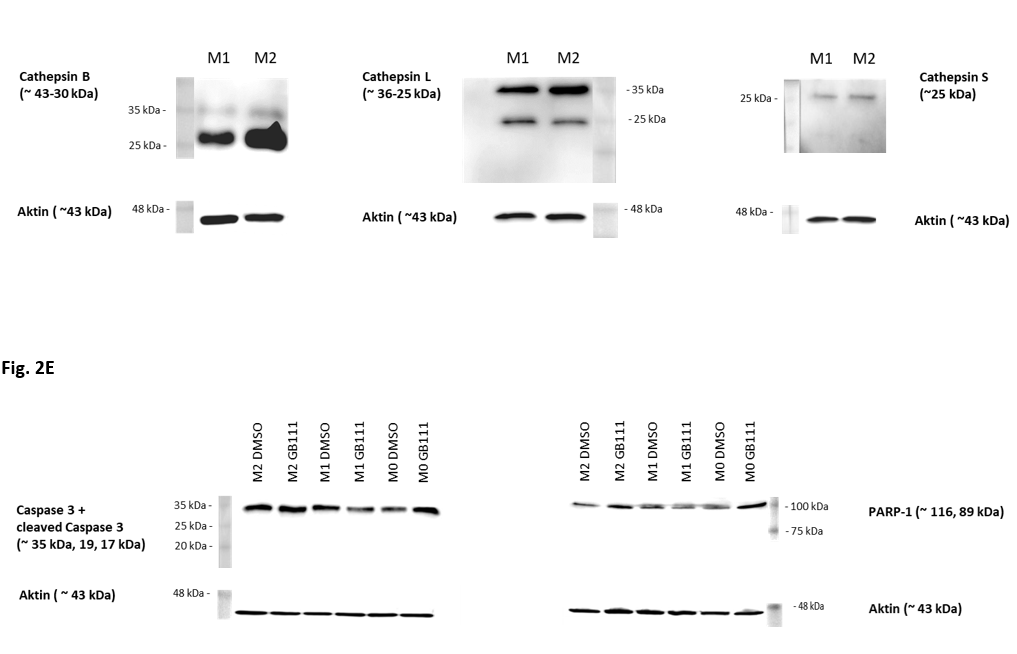


**Figure S2.** Uncropped immunoblots of the different macrophage populations with molecular weight markers.


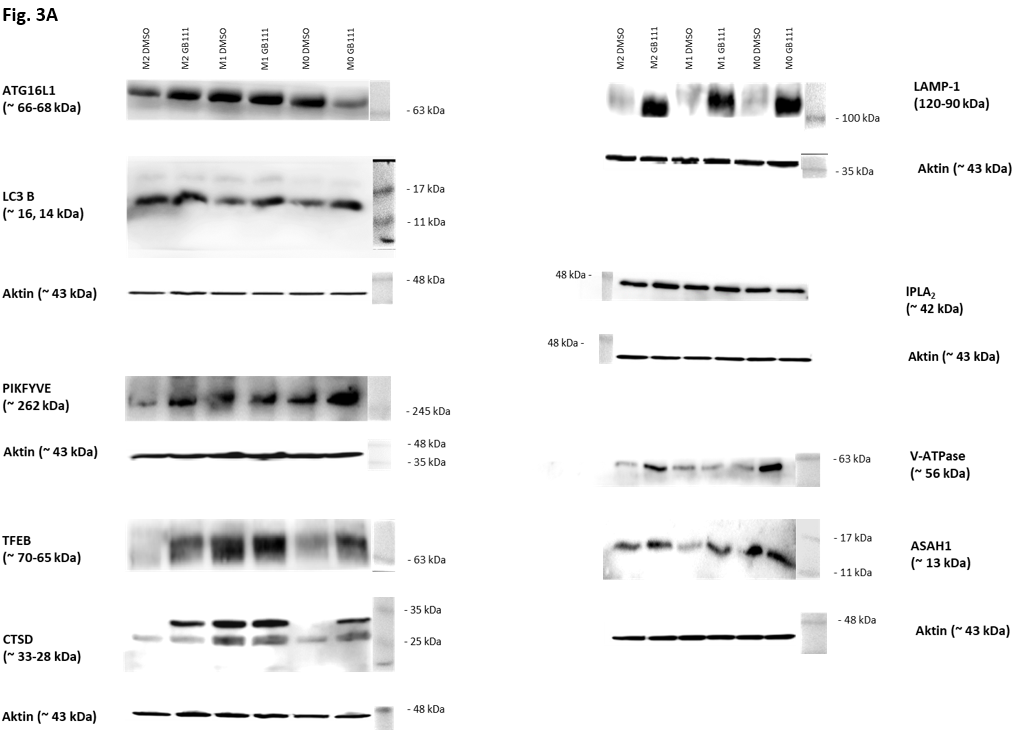


**Figure S3.** Uncropped immunoblots of the different macrophage populations with molecular weight markers.


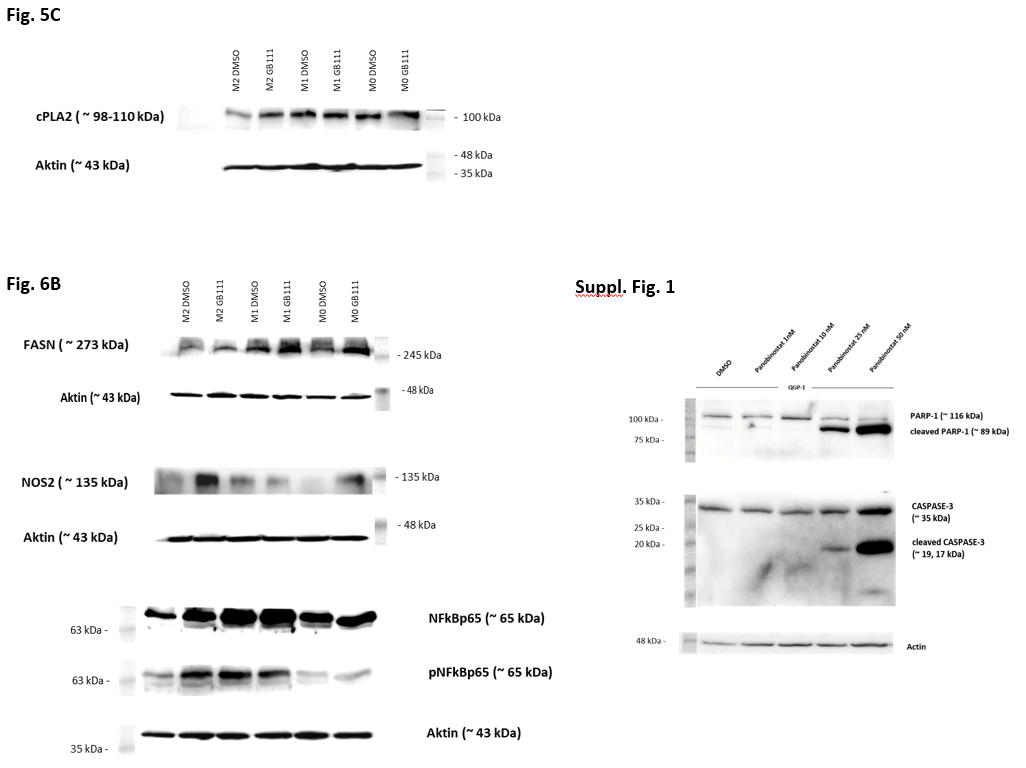


**Figure S4**. Uncropped immunoblots of the different macrophage populations with molecular weight markers.

**Table S1.** List of all primer pairs used for real-time PCR analysis with human and murine cells.

|  |  |
| --- | --- |
| **CCR7** | (s) AGTCTTCCAGCTGCCCTACA, (as) TCGTAGGCGATGTTGAGTTG |
| **CD206** | (s) GTGGCCGGAGTAGTCATCAT, (as) TCTTGAGGTAGGTGCACACG |
| **COX-2** | (s) GGTTGAATGTTTGTCCTTAGGATAGG, (as) CCACAGATCCCTCAAAACATTTT |
| **cPLA2** | (s) GAGTTTTGGGCGTTTCTGGT, (as) TCATCATCACTGTCCGAGCT |
| **FASN** | (s) TTCCGAGATTCCATCCTACG, (as) TGTCATCAAAGGTGCTCTCG |
| **IL-1α** | s) GCTACCAAAAAGACTCTACCCATATTACA, (as) GGTCTGTGGCTTCTTGTTTCAAC |
| **IL-1β** | (s) GCCAATCTTCATTGCTCAAGTGT, (as) AGCCATCATTTCACTGGCGA |
| **IL-6** | (s) TCCTGCAGAAAAAGGCAAAGA, (as) CATTTGTGGTTGGGTCAGGG; |
| **CCL2** | (s) AACCCAAGAATCTGCAGCTAACTT, (as) AAGGCATAATGTTTCACATCAACAA |
| **NOS2** | (s) ACCTTTGATGAGGGGACTGG, (as) GTTCTTCACTGTGGGGCTTG |
| **PGES2** | (s) GACAGCAGGACGGTTTGTTT, (as) TTGCAAACATGTCCCAGTCC |
| **RPLP0** | (s) GTCGGAGGAGTCGGACGAG, (as) GCCTTTATTTCCTTGTTTTGCAAA |
| **TFEB** | (s) TGGTGGAGATTCCCTGTCTT, (as) CAGGACCAGTTGCCTCAGAT |
| **TGFβ** | (s) CCTTTCCTGCTTCTCATGGC, (as) TCCGTGGAGCTGAAGCAATA |
| **TNFα** | (s) CCCATGTTGTAGCAAACCCT, (as) TTATCTCTCAGCTCCACGCC |
| **mCtsb** | (s) TTCCACCGGTCTCAACTACA, (as) TCACTGGCTATGCGAGGTAG |
| **mCtsl** | (s) AGGGTGACATGGTACAGCAA, (as) GACACTGAGGTGAAATCCGA |
| **mRplp0** | (s) TGGGCAAGAACACCATGATG, (as) AGTTTCTCCAGAGCTGGGTTGT |
